# Supplementary material for: Predicting pain location from resting-state brain fMRI
Source: bioRxiv. 2026 Jun 18:2026.06.14.732139. Preprint. [Version 1] doi: 10.64898/2026.06.14.732139 (PMC13307932; doi:10.64898/2026.06.14.732139)
Supplement: Supplement 1 [file NIHPP2026.06.14.732139v1-supplement-1.pdf]

bioRxiv preprint doi: https://doi.org/10.64898/2026.06.14.732139; this version posted June 18, 2026. The copyright holder for this preprint (which was not certified by peer review) is the author/funder, who has granted bioRxiv a license to display the preprint in perpetuity. It is made available under a CC-BY 4.0 International license.

Appendix A Body map dimensionality reduction

Community detection applied to the weighted covariance matrix of gridded pixel count data yielded a solution of 10 communities corresponding to distinct body parts, with an optimized weighting factor $\alpha = 0.90$ and a peak modularity = 0.71. These body parts are illustrated in Figure 5. Five of these communities encompassed grid sections from both the front and back body maps (head, left arm, right arm, left leg, and right leg) while five were restricted to either the front body (front chest, front pelvis) or back body (upper back, mid back, low back).

For each sensation, the mean normalized pixel count was calculated across all sections within each body part, yielding a vector of size 30 (number of sensations x number of body parts) for each subject. This matrix was then used as input to the NMF algorithm. The optimal number of factors was empirically selected to be 7 and confirmed with visual inspection of the cross-validation error curve. The resulting patterns are represented in Figure 5 of the main text.

The NMF reconstruction explained 79.60% of the variance of the input data. Pattern 1 explained a majority of the variance (29.25%) and represents low back pain predominantly (88.37% feature weight) with some front pelvis pain (7.53%). All other

bioRxiv preprint doi: https://doi.org/10.64898/2026.06.14.732139; this version posted June 18, 2026. The copyright holder for this preprint (which was not certified by peer review) is the author/funder, who has granted bioRxiv a license to display the preprint in perpetuity. It is made available under a CC-BY 4.0 International license.

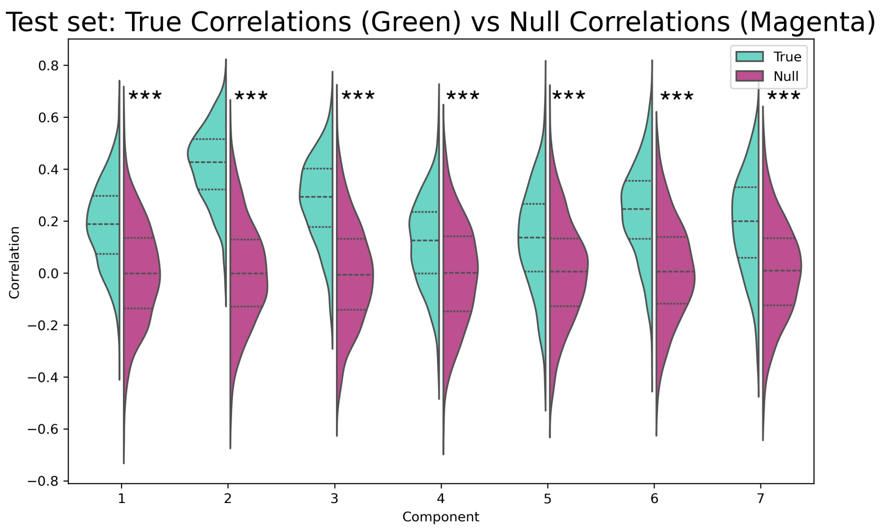

Fig. B1 Correlation of CCA components in true data vs. permuted data

patterns each contributed an additional 6 to 12% variance. Pattern 2 represents upper body pain, mostly upper back pain (58.22%) as well as some right arm pain (11.48%) and head pain (10.39%). Pattern 3 broadly represents arm symptoms, including left arm pain (22.05%), numbness (14.15%), and pins and needles (5.70%) as well as right arm numbness (11.34%) and pain (8.05%). Pattern 4 encompasses leg symptoms, including right leg pain (34.47%); pins and needles (9.11%) and left leg pain (22.17%); pins and needles (6.69%) as well as some front pelvis pain (8.60%) and right arm pain (5.56%). Pattern 5 is predominantly mid back pain (75.63%). Pattern 6 represents numbness and pins and needles throughout the back, with weights as follows: low back pins and needles (34.75%), low back numbness (21.92%), mid back numbness (6.75%), upper back pins and needles (5.16%), front pelvis pain (5.03%). Finally, Pattern 7 represents more leg symptoms, including left leg numbness (32.15%), pins and needles (10.61%) and pain (5.65%), and right leg numbness (25.13%) and pins and needles (5.26%).

Appendix B Permutation test for significance

Following the initial grid search, a 7-component solution was selected for further analysis, with an optimized $N_{CC} = 7$ in 40% of the grid search iterations. All 7 dimensions were significant in held-out test sets compared to shuffled data (Component 1: Mean $r = 0.19$, $p =$ 3.29e-110; Component 2: Mean $r = 0.41$, $p = 0$; Component 3: Mean $r = 0.28$, $p =$ 2.31e-215; Component 4: Mean $r = 0.12$, $p =$ 4.84e-48; Component 5: Mean $r = 0.13$, $p =$ 2.83e-47; Component 6: Mean $r = 0.24$, $p =$ 4.17e-145; Component 7: Mean $r = 0.19$, $p =$ 4.49e-95). Violin plots showing true and null correlations are presented in Figure B1.

bioRxiv preprint doi: https://doi.org/10.64898/2026.06.14.732139; this version posted June 18, 2026. The copyright holder for this preprint (which was not certified by peer review) is the author/funder, who has granted bioRxiv a license to display the preprint in perpetuity. It is made available under a CC-BY 4.0 International license.

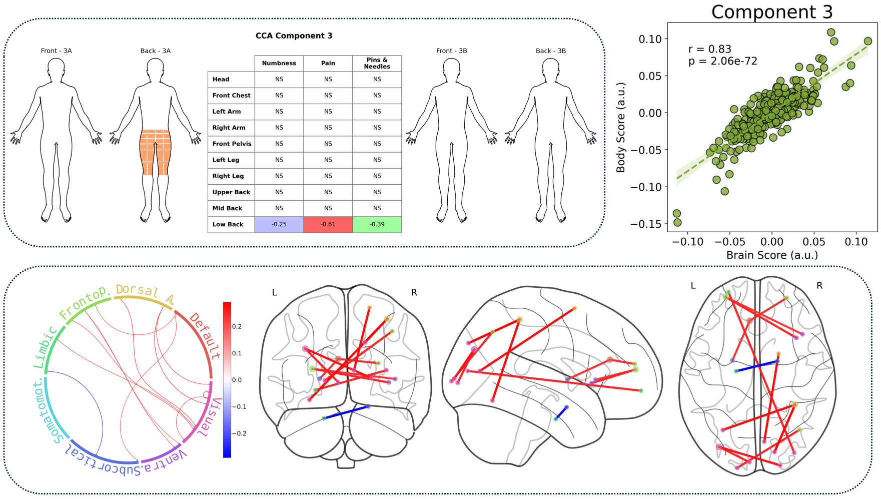

Fig. C2 Brain and body feature weights for Component 3.

Appendix C Additional Canonical Component Weights

Phenotype 3A (Figure C2) represents low back symptoms, with low back pain, numbness, and pins and needles all negatively correlated with this component. This phenotype shows stronger connectivity between the amygdala and parahippocampal gyrus, and weaker cortico-cortical connectivity between regions throughout the frontal, parietal, occipital, and cingulate cortices and the insula, as well as between the basal ganglia and the cingulate.

The fourth dimension (Figure C3) represents a superior-inferior gradient, with phenotype 4A showing upper body symptoms, including pain and numbness in the arms and upper to mid back, and 4B showing low body symptoms, such as low back pain and leg numbness and pins and needles. Phenotype 4A is associated with stronger connectivity between somatomotor regions, including S1, and DMN, attention, and limbic regions throughout the cortex. Phenotype 4B shows stronger connectivity between the thalamus and superior frontal gyrus.

Phenotype 5A is associated with pain in the legs and pelvis, while 5B involves pain in the upper left back and numbness in the left leg (Figure C4). Phenotype 5A shows stronger connectivity within and between various somatomotor, attention, and default regions, as well as between the thalamus and parts of the occipital cortex. Phenotype 5B shows stronger interhemispheric connectivity in the superior temporal gyrus.

Phenotype 6A is associated with low back pain, while 6B shows pins and needles in the upper and lower back and pain in the mid back (Figure C5). Phenotype 6A shows stronger connectivity between visual regions, in particular the left and right lateral occipital cortex, as well as the cingulate and precuneus. Phenotype 6B shows stronger connectivity between the superior parietal lobule and parts of the lateral occipital cortex.

bioRxiv preprint doi: https://doi.org/10.64898/2026.06.14.732139; this version posted June 18, 2026. The copyright holder for this preprint (which was not certified by peer review) is the author/funder, who has granted bioRxiv a license to display the preprint in perpetuity. It is made available under a CC-BY 4.0 International license.

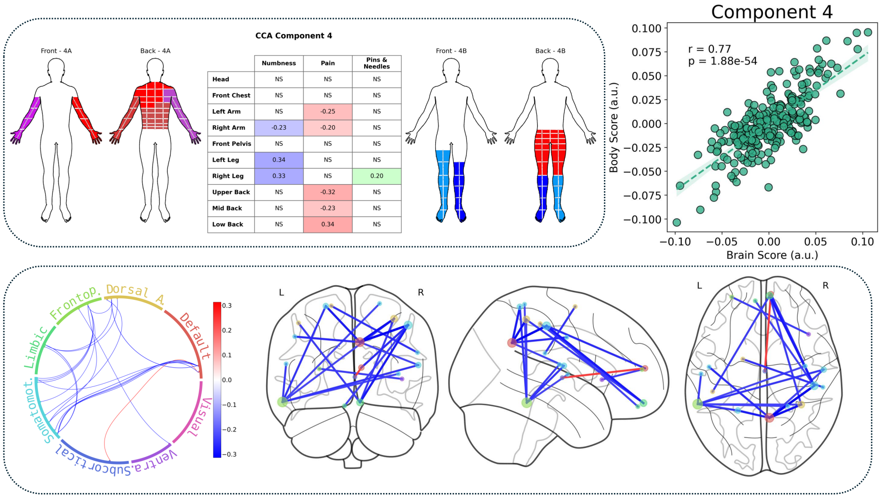

Fig. C3 Brain and body feature weights for Component 4.

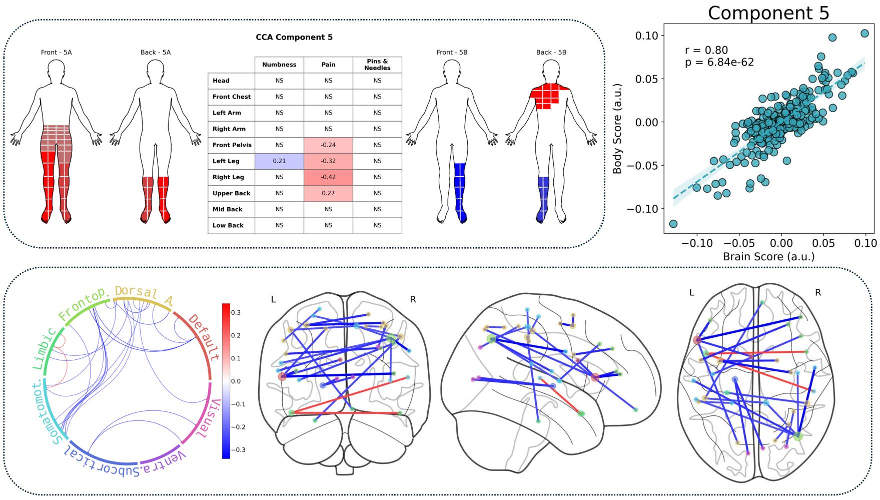

Fig. C4 Brain and body feature weights for Component 5.

Component 7 shows upper back pain on one end and mid back pain on the other. Phenotype 7B, the mid back pain phenotype, shows stronger connectivity between the left inferior frontal gyrus and the left insula, cingulate, and parahippocampal gyrus, as well as within the right parahippocampal gyrus.

bioRxiv preprint doi: https://doi.org/10.64898/2026.06.14.732139; this version posted June 18, 2026. The copyright holder for this preprint (which was not certified by peer review) is the author/funder, who has granted bioRxiv a license to display the preprint in perpetuity. It is made available under a CC-BY 4.0 International license.

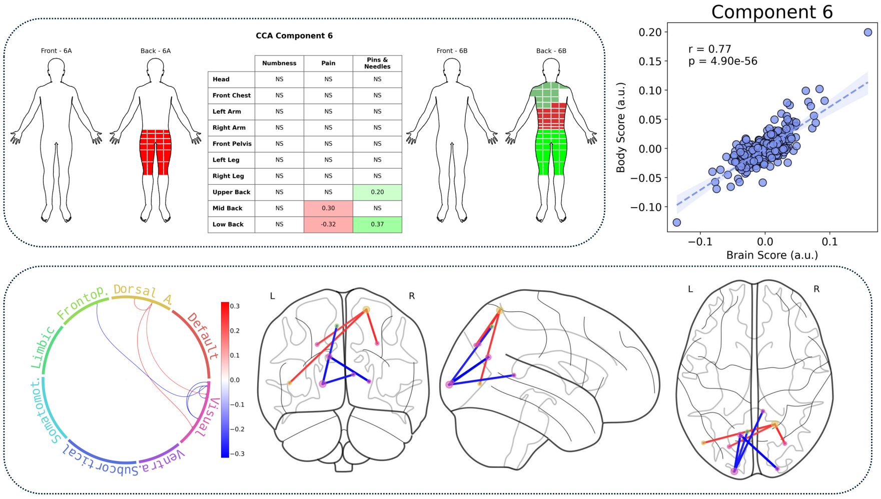

CCA Component 6

	Numbness	Pain	Pins & Needles
Head	NS	NS	NS
Front Chest	NS	NS	NS
Left Arm	NS	NS	NS
Right Arm	NS	NS	NS
Front Pelvis	NS	NS	NS
Left Leg	NS	NS	NS
Right Leg	NS	NS	NS
Upper Back	NS	NS	0.20
Mid Back	NS	0.30	NS
Low Back	NS	-0.32	0.37

Fig. C5 Brain and body feature weights for Component 6.

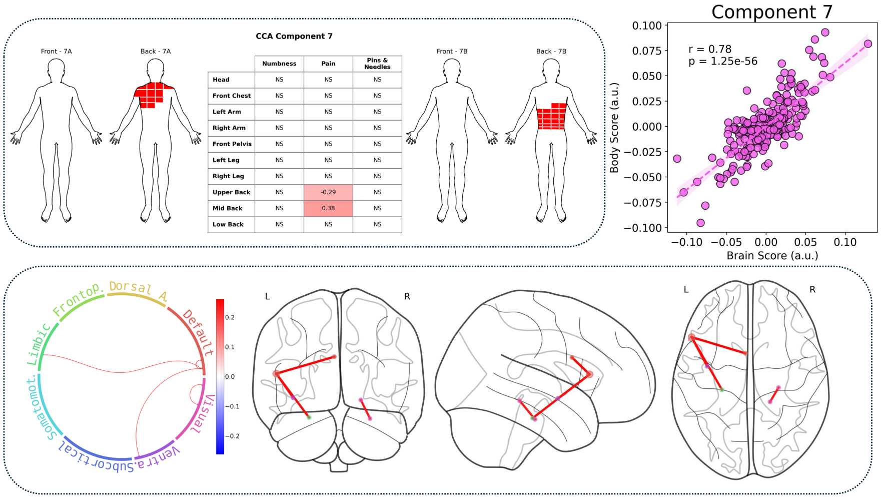

CCA Component 7

	Numbness	Pain	Pins & Needles
Head	NS	NS	NS
Front Chest	NS	NS	NS
Left Arm	NS	NS	NS
Right Arm	NS	NS	NS
Front Pelvis	NS	NS	NS
Left Leg	NS	NS	NS
Right Leg	NS	NS	NS
Upper Back	NS	-0.29	NS
Mid Back	NS	0.38	NS
Low Back	NS	NS	NS

Fig. C6 Brain and body feature weights for Component 7.

Appendix D Association with biopsychosocial factors

We investigated associations between each component score and a set of biopsychosocial risk and prognostic factors for LBP. Results are shown in Figures D7 - D9. Each column represents a component, and each row is a variable. Tests with $p \leq 0.05$ are indicated in the subplot title.

Component 1 is associated with almost all PROMIS variables with the exception of depression, suggesting that a tendency to report more bodily symptoms is also

bioRxiv preprint doi: https://doi.org/10.64898/2026.06.14.732139; this version posted June 18, 2026. The copyright holder for this preprint (which was not certified by peer review) is the author/funder, who has granted bioRxiv a license to display the preprint in perpetuity. It is made available under a CC-BY 4.0 International license.

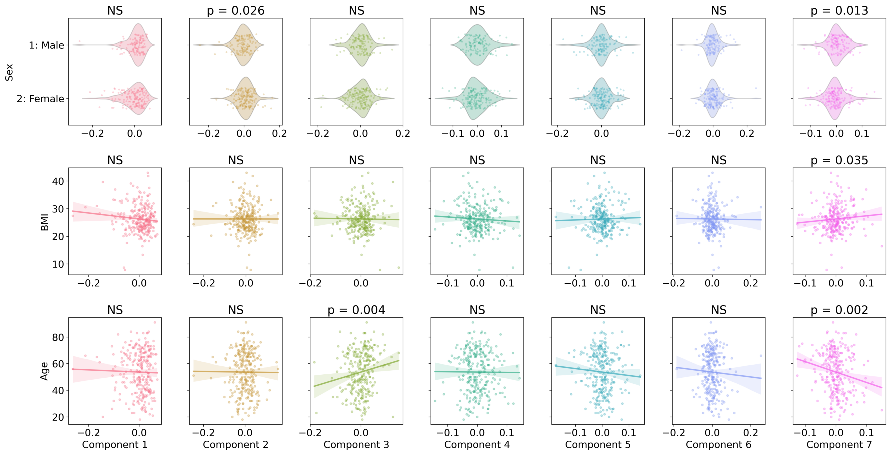

Fig. D7 Demographic Variables.

correlated with higher scores on negatively valanced PROMIS indicators (anxiety, fatigue, sleep disturbance) and lower scores on positively valanced PROMIS indicators (social roles and activities, cognitive function, physical function). Phenotype 1A is also associated with a higher PEG score and a higher prevalence of Modic Type 1 imaging findings. Component 2 showed an association with sex, with females tending more toward Phenotype 2B. Phenotype 3A is correlated with lower age and a higher score on the PROMIS fatigue scale. Component 7 is associated with several demographic factors, with Phenotype 7B correlated with female sex, higher BMI, and lower age. It is also associated with higher rates of social activity.

bioRxiv preprint doi: https://doi.org/10.64898/2026.06.14.732139; this version posted June 18, 2026. The copyright holder for this preprint (which was not certified by peer review) is the author/funder, who has granted bioRxiv a license to display the preprint in perpetuity. It is made available under a CC-BY 4.0 International license.

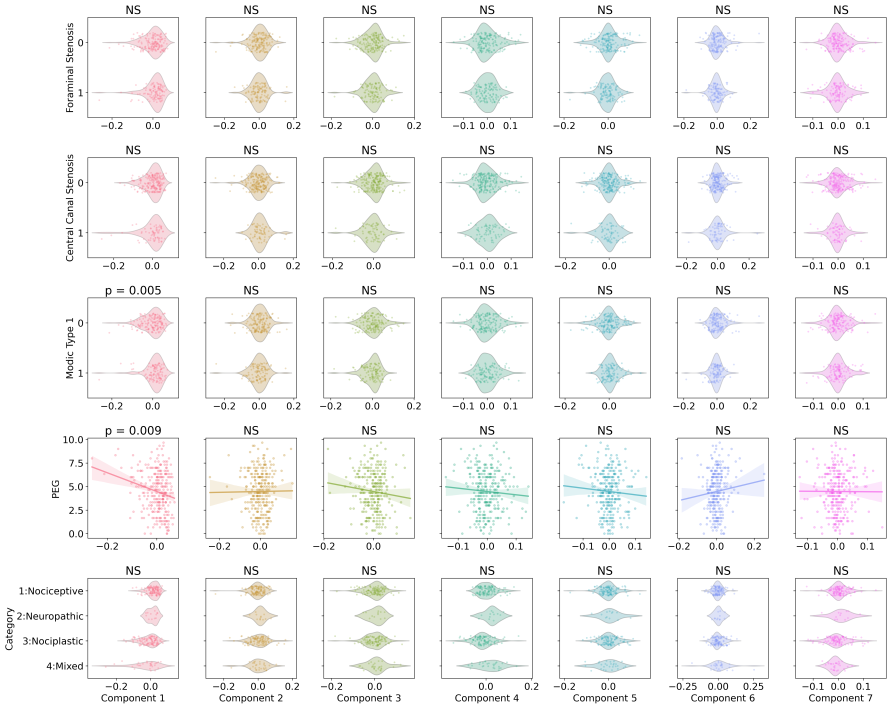

Fig. D8 Clinical Variables.

Appendix E fMRIPrep boilerplate text

Results included in this manuscript come from preprocessing performed using *fMRIPrep* 20.2.7 (Esteban et al (2018b); Esteban et al (2018a); RRID:SCR_016216), which is based on *Nipype* 1.7.0 (Gorgolewski et al (2011); Gorgolewski et al (2018); RRID:SCR_002502).

E.1 Anatomical data preprocessing

A total of 1 T1-weighted (T1w) images were found within the input BIDS dataset. The T1-weighted (T1w) image was corrected for intensity non-uniformity (INU) with `N4BiasFieldCorrection` Tustison et al (2010), distributed with ANTs 2.3.3 (Avants et al, 2008, RRID:SCR_004757), and used as T1w-reference throughout the workflow. The T1w-reference was then skull-stripped with a *Nipype* implementation of the `antsBrainExtraction.sh` workflow (from ANTs), using OASIS30ANTs as target template. Brain tissue segmentation of cerebrospinal fluid (CSF), white-matter (WM) and gray-matter (GM) was performed on the brain-extracted T1w using `fast` (FSL 5.0.9, RRID:SCR_002823, Zhang et al, 2001). Brain surfaces were reconstructed using `recon-all` (FreeSurfer 6.0.1, RRID:SCR_001847, Dale et al, 1999), and the brain mask estimated previously was refined with a custom variation of the method to reconcile ANTs-derived and FreeSurfer-derived segmentations of the cortical gray-matter of Mindboggle (RRID:SCR_002438, Klein et al, 2017). Volume-based spatial

bioRxiv preprint doi: https://doi.org/10.64898/2026.06.14.732139; this version posted June 18, 2026. The copyright holder for this preprint (which was not certified by peer review) is the author/funder, who has granted bioRxiv a license to display the preprint in perpetuity. It is made available under a CC-BY 4.0 International license.

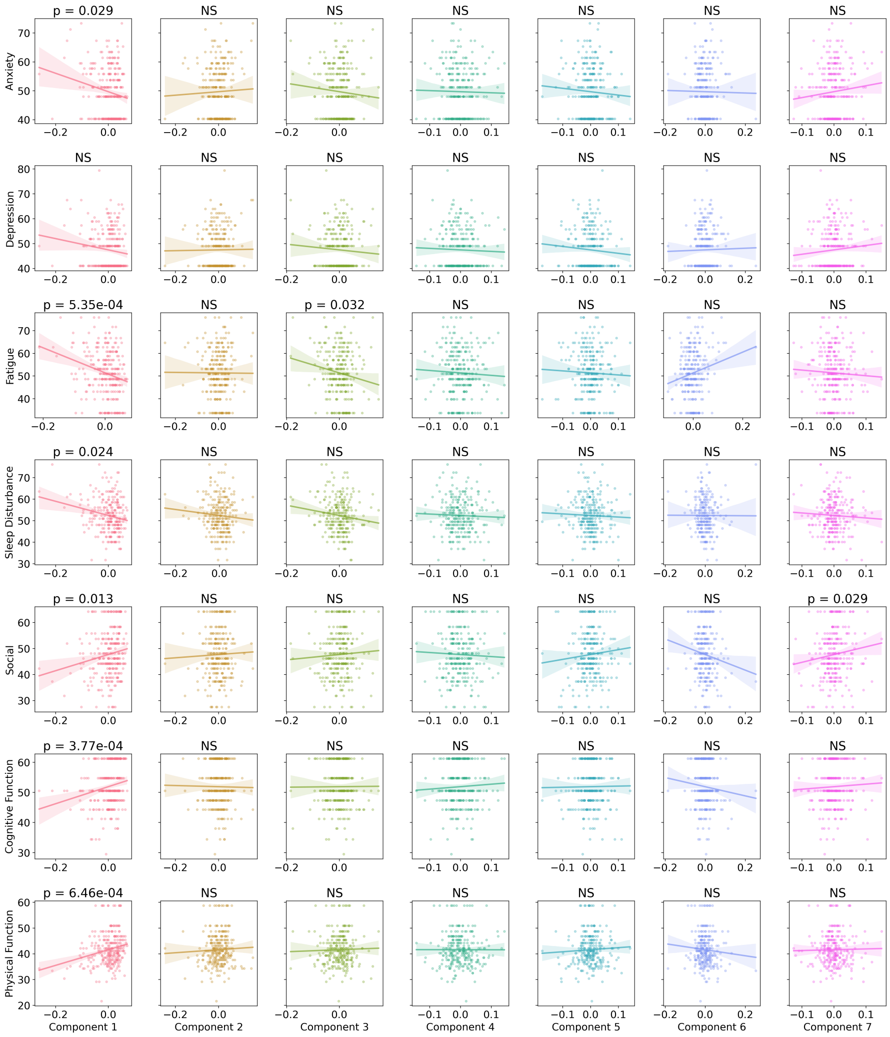

Fig. D9 Psychosocial Variables. Anxiety: PROMIS Anxiety 4a (higher = more anxiety); Depression: PROMIS Depression 4a (higher = more depressed); Fatigue: PROMIS Fatigue 4a (higher = more fatigued); Sleep Disturbance: PROMIS Sleep Disturbance (higher = more disturbance); Social: PROMIS Social Roles and Activities 4a (higher = more social); Cognitive Function: PROMIS Cognitive Function 2a (higher = more cognitive function), Physical Function: PROMIS Physical Function 6b (higher = more function).

normalization to two standard spaces (MNI152NLin2009cAsym, MNI152NLin6Asym) was performed through nonlinear registration with `antsRegistration` (ANTs 2.3.3), using brain-extracted versions of both T1w reference and the T1w template. The following templates were selected for spatial normalization: *ICBM 152 Nonlinear Asymmetrical template version 2009c* Fonov et al (2009), RRID:SCR_008796; TemplateFlow ID: MNI152NLin2009cAsym, *FSL's MNI ICBM 152 non-linear 6th*

bioRxiv preprint doi: https://doi.org/10.64898/2026.06.14.732139; this version posted June 18, 2026. The copyright holder for this preprint (which was not certified by peer review) is the author/funder, who has granted bioRxiv a license to display the preprint in perpetuity. It is made available under a CC-BY 4.0 International license.

Generation Asymmetric Average Brain Stereotaxic Registration Model Evans et al (2012), RRID:SCR_002823; TemplateFlow ID: MNI152NLin6Asym.

E.2 Functional data preprocessing

For each of the 1 BOLD runs found per subject (across all tasks and sessions), the following preprocessing was performed. First, a reference volume and its skull-stripped version were generated by aligning and averaging 1 single-band references (SBRefs). A deformation field to correct for susceptibility distortions was estimated based on *fMRIPrep*'s *fieldmap-less* approach. The deformation field is that resulting from co-registering the BOLD reference to the same-subject T1w-reference with its intensity inverted Wang et al (2017); Huntenburg (2014). Registration is performed with `antsRegistration` (ANTs 2.3.3), and the process regularized by constraining deformation to be nonzero only along the phase-encoding direction, and modulated with an average fieldmap template Treiber et al (2016). Based on the estimated susceptibility distortion, a corrected EPI (echo-planar imaging) reference was calculated for a more accurate co-registration with the anatomical reference. The BOLD reference was then co-registered to the T1w reference using `bbregister` (FreeSurfer) which implements boundary-based registration Greve and Fischl (2009). Co-registration was configured with nine degrees of freedom to account for distortions remaining in the BOLD reference. Head-motion parameters with respect to the BOLD reference (transformation matrices, and six corresponding rotation and translation parameters) are estimated before any spatiotemporal filtering using `mcflirt` (FSL 5.0.9, Jenkinson et al, 2002). BOLD runs were slice-time corrected to 0.351s (0.5 of slice acquisition range 0s-0.703s) using `3dTshift` from AFNI 20160207 (Cox and Hyde, 1997, RRID:SCR_005927). First, a reference volume and its skull-stripped version were generated using a custom methodology of *fMRIPrep*. The BOLD time-series (including slice-timing correction when applied) were resampled onto their original, native space by applying a single, composite transform to correct for head-motion and susceptibility distortions. These resampled BOLD time-series will be referred to as *preprocessed BOLD in original space*, or just *preprocessed BOLD*. The BOLD time-series were resampled into several standard spaces, correspondingly generating the following *spatially-normalized, preprocessed BOLD runs*: MNI152NLin2009cAsym, MNI152NLin6Asym. First, a reference volume and its skull-stripped version were generated using a custom methodology of *fMRIPrep*. Automatic removal of motion artifacts using independent component analysis (ICA-AROMA, Pruim et al, 2015) was performed on the *preprocessed BOLD on MNI space* time-series after removal of non-steady state volumes and spatial smoothing with an isotropic, Gaussian kernel of 6mm FWHM (full-width half-maximum). Corresponding "non-aggresively" denoised runs were produced after such smoothing. Additionally, the "aggressive" noise-regressors were collected and placed in the corresponding confounds file. Several confounding time-series were calculated based on the *preprocessed BOLD*: framewise displacement (FD), DVARS and three region-wise global signals. FD was computed using two formulations following Power (absolute sum of relative motions, Power et al (2014)) and Jenkinson (relative root mean square displacement between affines, Jenkinson et al

bioRxiv preprint doi: https://doi.org/10.64898/2026.06.14.732139; this version posted June 18, 2026. The copyright holder for this preprint (which was not certified by peer review) is the author/funder, who has granted bioRxiv a license to display the preprint in perpetuity. It is made available under a CC-BY 4.0 International license.

(2002)). FD and DVARS are calculated for each functional run, both using their implementations in *Nipype* (following the definitions by Power et al, 2014). The three global signals are extracted within the CSF, the WM, and the whole-brain masks. Additionally, a set of physiological regressors were extracted to allow for component-based noise correction (*CompCor*, Behzadi et al, 2007). Principal components are estimated after high-pass filtering the *preprocessed BOLD* time-series (using a discrete cosine filter with 128s cut-off) for the two *CompCor* variants: temporal (tCompCor) and anatomical (aCompCor). tCompCor components are then calculated from the top 2% variable voxels within the brain mask. For aCompCor, three probabilistic masks (CSF, WM and combined CSF+WM) are generated in anatomical space. The implementation differs from that of Behzadi et al. in that instead of eroding the masks by 2 pixels on BOLD space, the aCompCor masks are subtracted a mask of pixels that likely contain a volume fraction of GM. This mask is obtained by dilating a GM mask extracted from the FreeSurfer's *aseg* segmentation, and it ensures components are not extracted from voxels containing a minimal fraction of GM. Finally, these masks are resampled into BOLD space and binarized by thresholding at 0.99 (as in the original implementation). Components are also calculated separately within the WM and CSF masks. For each CompCor decomposition, the k components with the largest singular values are retained, such that the retained components' time series are sufficient to explain 50 percent of variance across the nuisance mask (CSF, WM, combined, or temporal). The remaining components are dropped from consideration. The head-motion estimates calculated in the correction step were also placed within the corresponding confounds file. The confound time series derived from head motion estimates and global signals were expanded with the inclusion of temporal derivatives and quadratic terms for each Satterthwaite et al (2013). Frames that exceeded a threshold of 0.5 mm FD or 1.5 standardised DVARS were annotated as motion outliers. All resamplings can be performed with *a single interpolation step* by composing all the pertinent transformations (i.e. head-motion transform matrices, susceptibility distortion correction when available, and co-registrations to anatomical and output spaces). Gridded (volumetric) resamplings were performed using `antsApplyTransforms` (ANTs), configured with Lanczos interpolation to minimize the smoothing effects of other kernels Lanczos (1964). Non-gridded (surface) resamplings were performed using `mri_vol2surf` (FreeSurfer).
